# National Health Insurance Notes

**Published:** 1923-10

**Authors:** 


### NATIONAL HEALTH INSURANCE NOTES.

### THE SOCIETIES DO NOT WANT CONTROL.

IN a recent editorial article in the *National Insurance Gazette*, the medical correspondent of the *Times* is handled satirically for "reviving the old statement that the Approved Societies are anxious to have some control over the doctors." The *Gazette* confirms the view expressed more than once in these columns that the societies have no such wish. It says:—

We have talked to a good many approved society leaders, but we have not yet discovered one who wants to control the medical profession or any part of it. Even if the negotiations were entirely in the hands of approved societies, that is, if the Minister washed his hands entirely after announcing the figure which he had available, there is no thought in the minds of approved society leaders, so far as we know, that they would want to exercise any control whatever.

### "WHAT THE SOCIETIES REALLY WANT."

Under this sub-heading in the same article we read on with some interest:—

There is no doubt that approved societies would like to have some speeding up in the proceedings for bringing a defaulting doctor more quickly to book. This doctor at the present time has too long a run, and he can give a poor and grudging service for a very long time, almost with impunity. He can give indifferent service to the public, and he can sign certificates with the most careless disregard to the approved society's rights. That is the kind of thing which approved societies might want to control, but it is the only kind of control which we have ever heard them speak of.

These are all things for dealing with which at the present time there is effective machinery. It rests, as we have so often pointed out, with the approved societies to see that the machinery is put in motion more frequently. That machinery can doubtless be improved, and the *National Insurance Gazette* would be rendering a real service if it would indicate in detail where the defects lie and what particular improvements are called for. The Consultative Council, embodying every type of society experience, has had abundant opportunities of putting forward proposals, and has found it necessary to suggest very little in the way of constructive suggestion. Therefore, it is not sufficient for our contemporary to say that the societies "want to urge the doctors forward; to weed out the black sheep; and, generally to get a square deal." The question is, How?

### HOW NOT TO GET A GOOD SERVICE.

In reply to our enquiry, we should probably be told that there is not so much in question the need for altered machinery as for a better disposition on the part of the doctors, and a better attitude of mind towards their panel work. But that is just what the best amongst the doctors in the Insurance Medical Service are working for, as the societies themselves would at once admit. And the way not to get a better service, but a worse one, is the way which the *Gazette* itself indicates in the front page article in the same issue as that from which we have already quoted. After indicating that there is 7s. 3d. available for payment of the doctors after January 1 next (as against the 9s. 6d. now paid), and that any Treasury Grants are out of the question, the *Gazette* advises the societies "to refuse to part with a single copper." If this is merely a means of stimulating the societies to issue a counterblast to the doctors' memorandum, it need not be seriously criticised, but if it means that the doctors, in the view of those competent to express society opinion, ought to be put back at once to the 1912 figure, that, we say, is not the way to get an improved service.

### A MISCHIEVOUS SUGGESTION.

In the report of an interview with the *Daily News*, the Secretary of the Hearts of Oak Benefit Society, Mr. James P. Lewis, is made to say that the amount allowed to approved societies for administrative purposes is to be reduced by 5d. per member, and probably that sum will go to the doctors. We hope that Mr. Lewis has been incorrectly reported, but it is not the first time that such a mischievous suggestion has been made. It is difficult to think of any proposal in connection with the question of the doctors' pay more calculated to exasperate society feeling, and it is safe to say that such a factor will not find any place in the Minister's calculations.

### WOEFUL INACCURACY.

Why is it that critics who set out to reform or repeal the panel system take so little trouble to ascertain the facts? Here is Dr. Blackhall-Morison, writing in the *English Review* on "The Failure of the Panel System and the Remedy," stating that "if the terms of the Act be examined," it will be found that a doctor, "to be on the panel at all, must convince a representative of the Ministry of Health that he, presumably a fully qualified practitioner and amenable to the discipline of his College and that of the General Medical Council, must have the *imprimatur* of the Ministry of Health, playing the part of a German Staats examiner, which permits an Insurance Committee to permit a panel doctor to see the panel patient." This is simply untrue, and the writer should examine the Act again, or, more simply, ask any one of the 12,000 doctors engaged in insurance practice what examination at the hands of the Ministry of Health he had to undergo before going on to the panel. The answer is, of course, that there is no such examination or enquiry of any kind. Whether there might not well be something of this kind to secure a theoretically more efficient service is another matter. Such a woefully inaccurate statement on a fundamental point rather discounts the value of other statements in the article, on many of which Dr. Blackhall-Morison is, in fact, misinformed.

### INSURING APPRENTICES.

An interesting question has just been considered by the Scottish Courts in a test case brought by the Society of Accountants in Edinburgh. Where there is a contract of apprenticeship, the apprentice is insurable if he receives a money payment. The substantial point in the case was whether small payments during the five years' apprenticeship were not, in effect, the return of the premium originally paid, so that there was in effect no money payment. The Courts held that it was not permissible to slump the whole transaction.

